# CO_2_ permeability of the rat erythrocyte membrane and its inhibition

**DOI:** 10.1080/14756366.2021.1952194

**Published:** 2021-07-14

**Authors:** Samer Al-Samir, Maximilian Prill, Claudiu T. Supuran, Gerolf Gros, Volker Endeward

**Affiliations:** a AG Vegetative Physiologie 4220, Zentrum Physiologie, Medizinische Hochschule Hannover, Hannover, Germany; b Neurofarba Department, Section of Pharmaceutical and Nutritional Sciences, University of Florence, Florence, Italy

**Keywords:** Rat erythrocyte, membrane CO_2_ permeability, membrane HCO_3_^−^ permeability, intraerythrocytic carbonic anhydrase activity, ^18^O exchange mass spectrometry

## Abstract

We have studied the CO_2_ permeability of the erythrocyte membrane of the rat using a mass spectrometric method that employs ^18 ^O-labelled CO_2_. The method yields, in addition, the intraerythrocytic carbonic anhydrase activity and the membrane HCO_3_^−^ permeability. For normal rat erythrocytes, we find at 37 °C a CO_2_ permeability of 0.078 ± 0.015 cm/s, an intracellular carbonic anhydrase activity of 64,100, and a bicarbonate permeability of 2.1 × 10^−3 ^cm/s. We studied whether the rat erythrocyte membrane possesses protein CO_2_ channels similar to the human red cell membrane by applying the potential CO_2_ channel inhibitors pCMBS, Dibac, phloretin, and DIDS. Phloretin and DIDS were able to reduce the CO_2_ permeability by up to 50%. Since these effects cannot be attributed to the lipid part of the membrane, we conclude that the rat erythrocyte membrane is equipped with protein CO_2_ channels that are responsible for at least 50% of its CO_2_ permeability.

## Introduction

In 1998, Nakhoul et al. and Cooper and Boron^1^^,^^2^ have shown that the Xenopus oocyte membrane exhibits an increase in its permeability for CO_2_ upon the incorporation of aquaporin-1 (AQP1), and Forster et al.^3^ demonstrated that the CO_2_ permeability of the human red cell membrane decreases drastically under the exposure to 4,4′-diisothiocyanato-stilbene-2.2′-disulfonate (DIDS), which they interpreted as a highly effective inhibitory action of DIDS upon an as yet unidentified protein CO_2_ channel in the erythrocyte membrane. Thereafter, Endeward and co-workers used human red cells deficient in AQP1 or in the Rhesus-associated glycoprotein (RhAG), to show that about 90% of the CO_2_ permeability of the human red cell membrane is due to these two proteins acting as CO_2_ channels^4–6^. They concluded that AQP1 and RhAG contribute about equally to this channel-mediated CO_2_ pathway. Analogous studies have not been reported so far for any other mammalian species.

The aim of the present study was to investigate whether red blood cells of the rat are similarly equipped with a gas channel-mediated CO_2_ pathway. We chose the rat because this animal is relatively easily accessible to physiological studies assessing the exchange of CO_2_ *in vivo*. We used the previously described mass spectrometric method to determine the CO_2_ permeability of rat red cells in suspension^7^^,^^8^. The red cells were exposed to several potential gas channel inhibitors, especially DIDS, but also Bis(1,3-dibutylbarbituric acid)pentamethine oxonol, Dibac, to phloretin, and to Para-(chloromercuri)-benzenesulfonate, pCMBS. Two of these substances proved to be highly efficient inhibitors of the protein-mediated CO_2_ pathway across the red cell membrane of the rat. In addition, we report here the first estimates of the intraerythrocytic carbonic anhydrase activity under physiological conditions of 37 °C, pH 7.2, and a CO_2_ partial pressure of 40 mmHg.

## Methods

### Animals and blood samples

Blood samples were taken from 3 months old Lewis rats from the Central Animal Facility of the Hannover Medical School by tail venipuncture in accordance with local regulations for animal experimentation^9^. The blood was spun down at 5000 *g* for 20 min, plasma removed and cells washed three times in 0.9% NaCl. Haematocrit, cell count, and haemoglobin concentration were determined by standard techniques. Mean corpuscular volume (MCV) was ∼63 fl, which is in agreement with previous reports^10^^,^^11^. Rat erythrocyte surface area, which was needed in addition to mean corpuscular volume for calculation of P_CO2_ and P_HCO3_^−^, was estimated from an established relation between red cell area and volume^12^ to be 100 µm^2^. This may be compared to the published red cell surface areas published for mice and humans (90 µm^2^ or 147 µm^2^, respectively^13^). Neither of the transport inhibitors specified below and acting on membrane CO_2_ permeability, namely phloretin and DIDS, had a significant effect on MCV after an exposure period of ∼5 min; all MCV values varied between 62 and 65 fl. No spherocytes were observed either in controls or with inhibitors, all red blood cells exhibited the regular biconcave shape.

### Inhibitors

Any potential extracellular carbonic anhydrase activity resulting from red cell lysis that may occur during the mass spectrometric determination of P_CO2_ and P_HCO3_^−^ was inhibited by the addition of the extracellular carbonic anhydrase inhibitor FC5-208A (2,4,6-trimethyl-1-(4-sulfamoyl-phenyl)-pyridinium perchlorate salt)^14^ to the assay at a final concentration of 5·× 10^−5 ^M. Thus, it was ensured that no extracellular carbonic activity was present during the mass spectrometric experiment with dilute red cell suspensions. Inhibition of channel-mediated membrane CO_2_ permeability was attempted by the following chemicals: DIDS (4,4′-diisothiocyanato-stilbene-2.2′-disulfonate; Sigma-Aldrich, Seelze, Germany), which has previously been shown by us to be an efficient inhibitor of human red cell P_CO2_ as well as P_HCO3_^3^^,^^4^^,^^5^; DiBAC (bis(1,3-dibutylbarbituric acid)pentamethine oxonol; Invitrogen GmbH, Karlsruhe, Germany), which is an established inhibitor of the erythrocytic HCO_3_^−^–Cl^−^ exchanger^15^ but does not inhibit P_CO2_ in human red cells^4^; pCMBS (para-(chloromercuri)-benzenesulfonate; Toronto Research Chemicals, North York, Canada; C367750), an established inhibitor of the aquaporin-1 water^16^ and CO_2_^2^^,^^5^ channels; phloretin (Sigma-Aldrich, Merck KGaA, Darmstadt, Germany; P7912), which is known to inhibit red cell bicarbonate-chloride exchange besides the transport of several other substrates^17^.

### Determination of CO_2_ and HCO_3_^−^ permeabilities

We have previously reported how the CO_2_ permeability of plasma membranes can be determined for red cells or other cells in suspension using a mass spectrometric method^4^^,^^5^^,^^7^^,^^8^. In principle, cells are exposed to a solution of C^18^O^16^O/HC^18^O^16^O_2_^−^ that is labelled with ^18 ^O to a degree of 1%. In this solution, C^18^O^16^O and HC^18^O^16^O_2_^−^ react with water or H^+^, thereby transferring by a defined probability the label ^18 ^O from the CO_2_–HCO_3_^−^ pool into the much larger pool of water. This reaction is slow, but inside red cells due to their high carbonic anhydrase activity becomes much faster. The exchange of ^18 ^O from CO_2_–HCO_3_^−^ into the water pool causes a decay of the species C^18^O^16^O (mass 46), and we observe this decay vs. time after the start of the exposure of the cells to the solution. In a first rapid phase, the carbonic anhydrase-containing cells rapidly take up C^18^O^16^O. The kinetics of this process depends on the permeability of the membrane to CO_2_ and on the speed of the intracellular conversion of CO_2_ to HCO_3_^−^, that is, on intracellular carbonic anhydrase activity. The rate of disappearance of C^18^O^16^O from the extracellular fluid is followed by a mass spectrometer equipped with a special inlet system for fluids as first described by Itada and Forster^18^.

**Figure 1.**
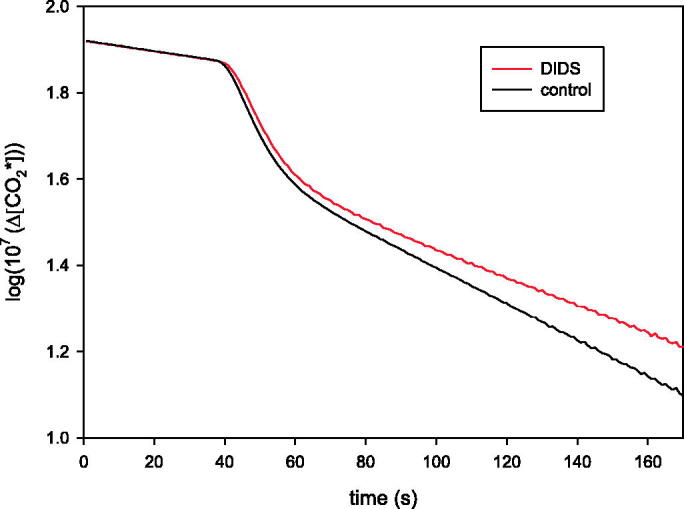
Time course of the decay of ^18 ^O in CO_2_ vs. time for rat red cells in the presence and absence of DIDS. Y-axis is log (10^7^·(Δ[CO_2_*])), where Δ[CO_2_*] is the concentration of ^18 ^O-labelled CO_2_ minus its final value at isotope equilibrium, in the unit 10^−7 ^M. The Y-axis gives the logarithm of this value after it has been multiplied by 10^7^. The curve shows three phases: (1) a pre-phase representing the slow uncatalysed decay of ^18^O-labelled CO_2_, (2) by adding, at the sharp bend in the curve, red cells into the measuring chamber the next phase is initiated, which we call the rapid first phase after addition of red cells and which is strongly dependent on P_CO2_, (3) a second slower phase follows, which is dependent on P_HCO3_^−^. During the mass spectrometric measurement of red cells an extracellular pH of 7.4 and a CO_2_ partial pressure of 40 mmHg prevail.

Examples are shown in Figure 1. From the time course of the rapid first phase of the disappearance of C^18^O^16^O (see Figure 1), the membrane permeability for CO_2_ can be calculated, if the intracellular carbonic anhydrase activity has been determined independently^7^. After the first rapid phase of the mass spectrometric record, a slower phase follows (also seen in Figure 1), which is to a major extent determined by the transport HC^18^O^16^O_2_^−^ across the membrane. Thus, this second phase allows one to determine membrane HCO_3_^−^ permeability^7^. For a complete review of the method see^8^.

To perform mass spectrometric measurements, a chamber with a volume of 2.2 ml was used that was attached to the high vacuum of the mass spectrometer via the previously published inlet system^7^. This chamber had a water jacket that kept the solutions at 37 °C and contained a magnetic stirrer mixing the content continuously. The solution in the chamber consisted of 110 mM NaCl, 20 mM HEPES and 25 mM NaHCO_3_ labelled with ^18 ^O to the extent of 1%. pH was adjusted to 7.4 and monitored during the entire measurement with a pH electrode. The reaction was started by adding 10 µl of red cell suspension with a haematocrit of 5% into the reaction chamber. In all experiments 5·× 10^−5 ^M FC5-208A was present. Red cell suspensions were incubated with this carbonic anhydrase inhibitor for 2–5 min before use in the mass spectrometer experiment. Likewise, if any of the above membrane transport inhibitors were used, they were incubated with red cells 2–5 min prior to the red cells’ addition into the mass spectrometer’s reaction chamber. For all inhibitors, it was ascertained that the same inhibitor concentration used for preincubation also existed in the bicarbonate buffer present in the reaction chamber. The same preincubation times for inhibitors have previously been used for human red cells^4^^,^^5^. Carbonic anhydrase activity was measured in suitably diluted lysed red cells, which gives in a plot like that of Figure 1 a mono- rather than a biphasic response^3^. The inhibitor-free lysates had a pH of 7.2 and a Cl^−^ concentration of 63 mM, mimicking the intraerythrocytic conditions. The acceleration factor describing the increase in slope caused by the addition of the lysate was used to calculate the intraerythrocyic carbonic anhydrase activity from (acceleration factor − 1) × dilution factor^3^.

## Results

### Intraerythrocytic carbonic anhydrase activity

Intraerythrocytic carbonic anhydrase activity (Ai) was determined at pH 7.2 from mass spectrometry of lysed red cells. Activity was determined for the blood sample from each animal studied, and used to calculate P_CO2_ and P_HCO3_^−^ from the data for this same blood’s red cells using the system of equations that describe the entire ^18 ^O-exchange process^7^. On average, Ai was 64,100 ± 6200 (SD; *n* = 31). Ai was defined as (acceleration factor − 1), where the acceleration factor is the factor by which the speed of CO_2_ hydration in the undiluted red cell interior was accelerated over the uncatalysed rate.

### Membrane CO_2_ permeability

**Figure 2.**
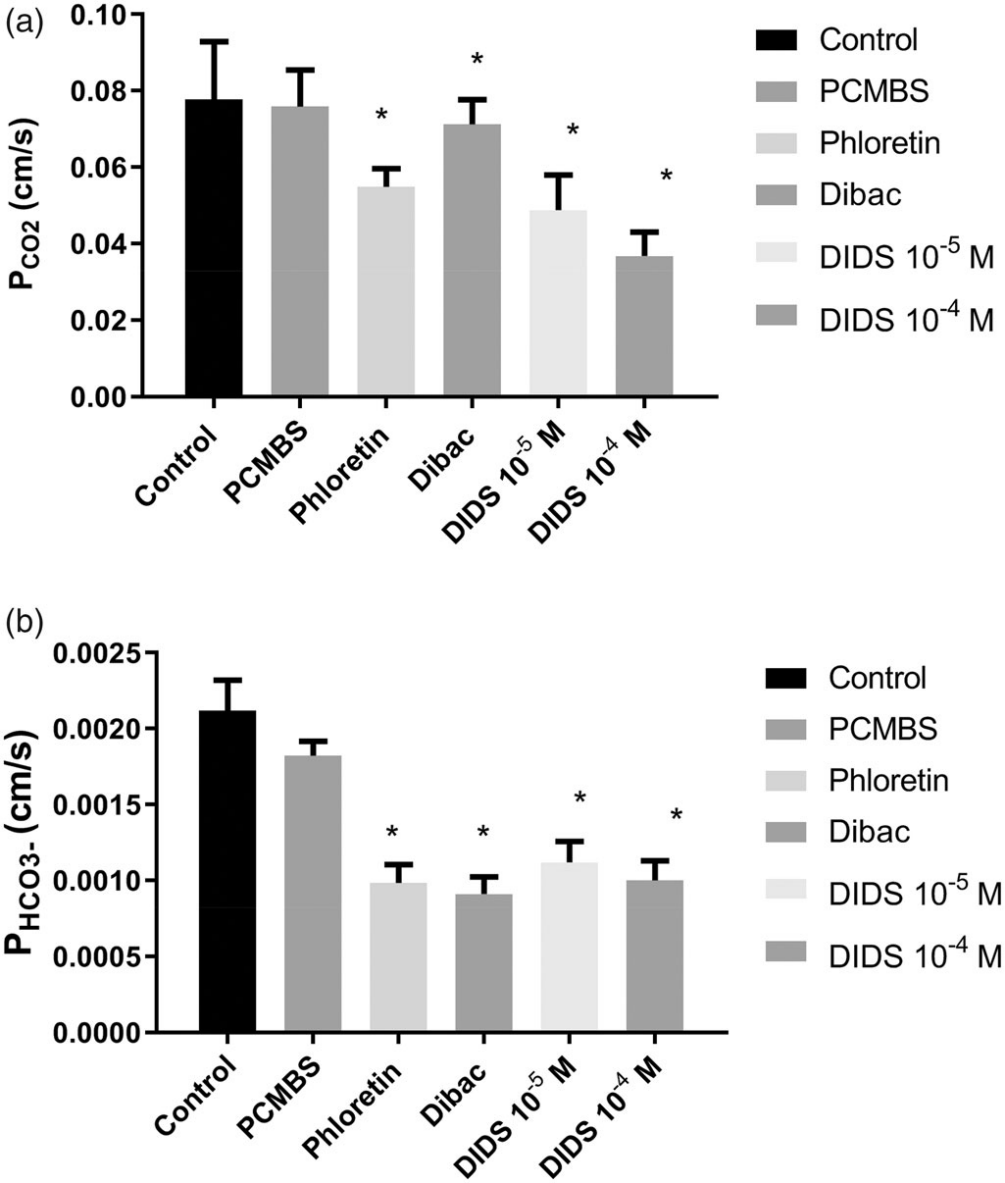
(a) CO_2_ permeability of rat red cells at 37 °C and effect of four inhibitors. Number of blood samples *n* from left to right: 36, 9, 8, 8, 10, 10. *Indicates statistical significance of difference to control (one-way ANOVA followed by Dunnett’s post-test). Inhibitor concentrations given are in mol/l. pCMBS was used at a concentration of 1 mM, phloretin at 300 µM, and Dibac at 5× 10^−7 ^M. (b) HCO_3_^−^ permeability of rat red cells at 37 °C and effect of four inhibitors. n from left to right: 36, 8, 8, 8, 10, 10. *Indicates statistical significance of difference to control (one-way ANOVA followed by Dunnett’s post-test). pCMBS was used at a concentration of 1 mM, phloretin at 300 µM, and Dibac at 5× 10^−7 ^M.

Figure 2(a) shows the results for P_CO2_ of the rat erythrocyte membrane at 37 °C. The control value in the absence of potential gas channel inhibitors is 0.078 ± 0.015 cm/s (SD; *n* = 36; left-hand column). The other columns of Figure 2(a) show the effect of various inhibitors on P_CO2_. 300 µM phloretin and DIDS at concentrations both of 10^−5^ and 10^−4 ^M significantly reduce CO_2_ permeability. In the presence of 10^−4 ^M DIDS, P_CO2_ amounts to 0.037 ± 0.006 cm/s (*n* = 10), that is, to about 1/2 of the control value. 1 mM pCMBS has no effect on P_CO2_, and 5·× 10^−7 ^M Dibac, a rather specific inhibitor of the erythrocytic Cl^−^–HCO_3_^−^ exchanger AE1^15^, has a very minor, although statistically significant, effect on P_CO2_ (0.071 ± 0.006 cm/s; *n* = 8). In conclusion, two of the four inhibitors used reduce P_CO2_ to a major extent.

### Membrane HCO_3_^−^ permeability

Figure 2(b) shows the effects of the same group of inhibitors on P_HCO3_^−^. The control value of P_HCO3_^−^ amounts to 0.0021 ± 0.0002 cm/s (*n* = 36; left-hand column). 1 mM pCMBS has again no significant effect, but all other inhibitors including Dibac have major and statistically significant effects. With 5·× 10^−7 ^M Dibac, P_HCO3_^−^ is reduced to 0.00091 ± 0.00011 cm/s (*n* = 8), that is, to less than 1/2 of the control value.

## Discussion

### Red cell carbonic anhydrase activity in rats

We report here an intraerythrocytic carbonic anhydrase activity of 64,100, which may be compared with the value of 21,000 we obtained for human red cells with the same method under identical conditions^4^. Thus, rat red cells have a 3 times higher Ai compared to human red cells. While the absolute numbers of these activities cannot be compared with the results obtained by Larimer and Schmidt-Nielsen^19^ due to highly different methods and conditions, it may be noted that these authors similarly found a 2.5 times higher Ai in rats compared to humans. In general, these authors observed a tendency of Ai to increase with decreasing body weight in mammals. The functional significance of the higher Ai values in smaller animals is not clear. Larimer and Schmidt-Nielsen speculated that the high carbonic anhydrase activity in small animals with their higher specific rate of oxygen consumption may improve oxygen release from red cells by an accelerated production of H^+^ from CO_2_, thus accelerating the contribution of the Bohr effect to O_2_ release^19^. Their studies have been discussed and updated in a more recent review^20^.

### CO_2_ permeability of the red cell membrane

#### P_CO2_ value

We report here a P_CO2_ of red cells of the rat of 0.078 cm/s at 37 °C. This value is half of the value reported previously for human red cells, 0.15 cm/s^4^^,^^5^. It must be considered, on the other hand, that the surface-to-volume ratio of rat red cells is >10% greater than that of human red cells, and that the rat red cell thickness is estimated to be 10% less than that of human red cells, both factors accelerating the speed of uptake of gases by rat red cells. This may lead to a gas uptake kinetics that overall is similar in rat and human red cells. This corresponds to the similar lung capillary transit times that have been reported for both rats and humans^21^^,^^22^.

#### Effects of inhibitors and role of CO2 channels

None of the potential gas channel inhibitors studied here is expected to have an effect on the CO_2_ permeability of the pure phospholipid bilayer. It has been shown that DIDS has no effect on P_CO2_ of pure phospholipid vesicles and cell membranes without gas channels^23^^,^^24^, but lowers P_CO2_ in both types of membranes when gas channels are present^4^^,^^5^^,^^23^^,^^25^. Similarly, it has been shown that pCMBS does not affect the P_CO2_ of membranes in the absence of gas channels, but it does reduce P_CO2_ when the gas channel aquaporin 1 is present^2^^,^^5^. No such direct evidence is available for Dibac and phloretin, but there is overwhelming evidence in the literature that these inhibitors suppress specifically the transport activity of the anion exchanger AE1 (Dibac^15^) or affect a wide range of transport proteins (phloretin^17^), respectively, but not the phospholipid membrane per se. We conclude that both DIDS and phloretin reduce membrane P_CO2_ by acting on a protein CO_2_ channel present in the membrane of rat erythrocytes. The maximal inhibition of P_CO2_ achieved here amounts to 50% of the control P_CO2_. This suggests that at least 50% of the P_CO2_ of rat red cells is due to a gas channel. This is a little less than has been reported for normal human red cells, where P_CO2_ fell by DIDS to about 1/3 of the control value^5^^,^^6^.

Our previous work has shown that Dibac does not affect P_CO2_ in human erythrocytes, although it drastically reduces P_HCO3_^4^. This is not unexpected as Dibac is a highly specific inhibitor of AE1^15^. Both findings are thus in line with the present observation of no major inhibition of P_CO2_ in rat erythrocytes by Dibac. However, we find here also no effect of pCMBS on the P_CO2_ of rat red cells, while previously we found a moderate suppressive effect of 1 mM pCMBS on P_CO2_ of human red cells. Importantly, this effect was substantially less pronounced than the effect of DIDS^5^. It may be noted that neither DIDS nor pCMBS permeate the red cell membrane and thus cannot affect intraerythrocytic carbonic anhydrase^4^^,^^5^.

A reasonable hypothesis to explain these observations comes from the following considerations:

- In human red cells, there are two major CO_2_ channels, AQP1 and RhAG, that contribute about equally to the CO_2_ permeability of the membrane and together are responsible for about 90% of the total CO_2_ permeability^4^^,^^5^.
- It is clear that DIDS inhibits quite effectively both AQP1 and RhAG CO_2_ channels, perhaps RhAG even a little more markedly than AQP1^6^.
- pCMBS inhibits P_CO2_ of human red cells clearly less than does DIDS, and this inhibitory effect seems to be exclusively due to AQP1, because in AQP1-deficient human red cells, whose only CO_2_ channel is RhAG, an effect of pCMBS is not detectable^5^.
- According to molecular dynamics studies, the central pore of the aquaporin 1 tetramer is the major pathway of CO_2_ across aquaporin 1, and the water channel of the aquaporin 1 monomer is a minor CO_2_ pathway^26^^,^^27^.
- Thus, it seems likely that the inhibitory effect of pCMBS we see on P_CO2_ of human red cells is mainly due to inhibition of the water (and CO_2_) channel of monomeric AQP1. It is well known that pCMBS, by acting on this pathway, largely blocks its water as well as its CO_2_ permeability via binding to a cysteine in position 189 in the water pore^2^^,^^28^^,^^29^. pCMBS does not act on the RhAG CO_2_ pathway and likely does not act on the central pore of the aquaporin tetramer. It is probable that the properties of human AQP1 discussed under C) to E) apply analogously to rat AQP1.
- In rat erythrocytes it is well established that AQP1 as well as RhAG are present in the membrane^30–32^. Unfortunately, to our knowledge there is no quantitative information on the abundance of AQP1 and RhAG expression in rat red cells.

Overall we draw the following conclusions:

1. Rat, as well as human red cells, express CO_2_ channels in their membrane, which constitute a sizable part of the membrane CO_2_ permeability. These channels can very effectively be inhibited by DIDS and also, to a lesser extent, by phloretin. The effect of DIDS is qualitatively compatible with results obtained for human red cells, suggesting that AQP1 and RhAG represent the CO_2_ channels of rat erythrocytes. Likewise, the effect of phloretin reported here is similar to that observed in human red cells (own unpublished observations).
2. However, the fact that the effect of DIDS on P_CO2_ is smaller in comparison to human red cells, suggests that the role of CO_2_ channels in the rat may be somewhat less pronounced than it is in human red cells.
3. This would be in agreement with the present observation of a lack of an effect of pCMBS on P_CO2_, which might indicate that it is AQP1, which is expressed to a lesser degree in rat erythrocytes. However, conclusive evidence on this last point is lacking. An alternative explanation might a lower cholesterol content of the rat erythrocyte membrane, which would increase the membrane’s intrinsic permeability to CO_2_^23^ and thus render the effect of CO_2_ channels less visible. However, the cholesterol content of the rat erythrocyte membrane has not been reported, and also, the CO_2_ permeabilities seen here for rat red cells are lower than those for human red cells.
4. Instead, the lower P_CO2_ of rat red cells might – at least partially – be caused by the reduced role of CO_2_ channels in rats compared to human erythrocytes.

### HCO_3_^−^ permeability of the red cell membrane

Applying the present mass spectrometric technique, we have previously reported the bicarbonate permeability of normal human red blood cells to be 1.2–1.3 × 10^−3 ^cm/s at 37 °C^4–6^. The present control value for rat red cells is 2.1 × 10^−3 ^cm/s at the same temperature. This may be surprising in the view of the P_CO2_ that is lower for rat red cells than it is for human red cells (see above). It may be considered that in the case of HCO_3_^−^ uptake or release, the membrane constitutes the major diffusion resistance since P_HCO3_^−^ is almost 50 times lower than P_CO2_, and since the intracellular diffusion of HCO_3_^−^ in comparison is much faster (albeit almost equally fast as the intracellular diffusion of CO_2_). If then the product of P x cellular surface area is decisive for the kinetics of HCO_3_^−^, we would, with the numbers given by^13^ and above, obtain products of 1.25 × 10^−3 ^cm/s × 146 µm^2^ for humans and of 2.1 × 10^−3 ^cm/s × 100 µm^2^ for rats, respectively. In other words, these products agree within about 15%, and, as in the case of CO_2_ uptake kinetics, we thus expect similar kinetics of HCO_3_^−^ exchange in both rat and human red cells. The inhibitory profile of P_HCO3_^−^ seen in Figure 2(b) with strong effects of Dibac, phloretin and DIDS is as expected from the literature, as detailed in the Methods section.

### Outlook

We present clear evidence that the red cell membrane of the rat possesses protein channels for CO_2_, which can effectively be inhibited by DIDS and by phloretin. In studies on the role of gas channels for red cell CO_2_ exchange, the difficulty may arise that both these inhibitors inhibit CO_2_ permeation as well as HCO_3_^−^ permeation. Both these processes contribute to overall CO_2_ exchange, although with quite different kinetics. This problem can, at least *in vitro*, be solved by complementing studies with DIDS or phloretin with experiments using Dibac, which very efficiently inhibits P_HCO3_^−^ without affecting P_CO2_. This should allow one to dissect the effects from inhibition of P_CO2_ from those resulting from inhibition of P_HCO3_^−^.
